# Alternatively Spliced *BnaPAP2.A7* Isoforms Play Opposing Roles in Anthocyanin Biosynthesis of *Brassica napus* L.

**DOI:** 10.3389/fpls.2020.00983

**Published:** 2020-08-19

**Authors:** Daozong Chen, Yi Liu, Shuai Yin, Jie Qiu, Qingdong Jin, Graham J. King, Jing Wang, Xianhong Ge, Zaiyun Li

**Affiliations:** ^1^ National Key Laboratory of Crop Genetic Improvement, National Center of Oil Crop Improvement (Wuhan), College of Plant Science and Technology, Huazhong Agricultural University, Wuhan, China; ^2^ Southern Cross Plant Science, Southern Cross University, Lismore, NSW, Australia

**Keywords:** *Brassica napus* L., anthocyanin, R2R3-MYB, *BnaPAP2.A7*, alternative splicing

## Abstract

*Brassica napus* L. (rapeseed, oilseed rape, and canola) and varieties of its two diploid parents, *B. oleracea* and *B. rapa*, display a large amount of variation in anthocyanin pigmentation of the leaf, stem, and fruit. Here, we demonstrate that *BnaPAP2.A7*, an ortholog of the *B. oleracea* anthocyanin activator *BoMYB2* that confers purple traits, positively regulates anthocyanin biosynthesis in leaves of *B. napus*. Sequencing of *BnaPAP2.A7* and transgenic analysis suggests that activation of this gene in purple rapeseed may result from a single nucleotide and/or 2bp insertion in its promoter region. *BnaPAP2.A7* gives rise to three splice variants, designated *BnaPAP2.A7-744, BnaPAP2.A7-910*, and *BnaPAP2.A7-395* according to the length of the transcripts. While *BnaPAP2.A7-744* encodes a full-length R2R3-MYB, both *BnaPAP2.A7-910* and *BnaPAP2.A7-395* encode truncated proteins that lack both a partial R3 repeat and the complete C terminal domain, and so *in vitro* are unable to interact with the *Arabidopsis* bHLH protein AtTT8. Although expression of either *BnaPAP2.A7-910* or *BnaPAP2.A7-395* in green rapeseed does not result in purple leaves, both genes do modify genome-wide gene expression, with a strong repression of anthocyanin-related genes. We have demonstrated that *BnaPAP.A7* regulates anthocyanin accumulation in leaves of *B. napus* and propose a potential mechanism for modulation of anthocyanin biosynthesis by alternative splicing.

## Introduction

Anthocyanins are a group of plant secondary metabolite flavonoid pigments. They play the role of “nature’s Swiss army knife” in conferring tolerance to various stressors as well as in defense against herbivores and pathogens (Gould, 2004). Anthocyanins are biosynthesized from phenylalanine *via* a series of enzymes encoded by structural genes including phenylalanine-ammonia lyase (*PAL*), cinnamate 4-hydrodylase (*C4H*), and 4-coumaryl, CoA ligase (*4CL*), early anthocyanin biosynthesis (EBGs) genes, chalcone synthase (*CHS*), chalcone isomerase (*CHI*), and flavanone 3-hydroxylase (*F3H*), late anthocyanin genes (LBGs), dihydroflavonol 4-reductase (*DFR*), anthocyanidin synthase (*ANS*), and anthocyanidin 3-O-glucosyltransferase (*UFGT*) (Saito et al., 2013). The expression of these genes is usually regulated at the transcriptional level and primarily by individual MYB transcription factors (TFs). Of these, the MBW transcription factor complex, composed of R2R3-MYB, basic-helix-loop-helix (bHLH), and WD40 proteins, has been shown to control expression of the LBGs (Broun, 2005; Ramsay and Glover, 2005; Gonzalez et al., 2008; Petroni and Tonelli, 2011; Albert et al., 2014; Xu et al., 2015).

Within the MBW complex R2R3-MYBs are the major determinants involved in anthocyanin biosynthesis (Lai et al., 2014; Liu et al., 2015; Jin et al., 2016), either as activators or repressors of structural genes (Pérez-Díaz et al., 2016). They represent the largest subgroup of the plant MYB TF family, and they have two MYB repeats in the N terminal DNA-binding domain that are most similar to R2 and R3 from c-MYB (Feller et al., 2011) as well as an activation or repression domain usually located at the C terminus (Zimmermann et al., 2004; Dubos et al., 2010; Xu et al., 2014). Based on the conservation of the DNA binding domain and amino acid motifs in the C terminal domains, R2R3-MYB proteins have been classified into several subgroups that are involved in plant-specific processes (Dubos et al., 2010). *ZmC1* was the first R2R3-MYB transcription activator identified, reported to regulate the synthesis of anthocyanins in the aleurone of maize (*Zea mays*) kernels (Cone et al., 1986; Paz-Ares et al., 1987). *ZmPl* was later reported to regulate biosynthesis of anthocyanins that give rise to the purple leaf phenotype in maize (Cone et al., 1993). R2R3-MYB transcription activators have subsequently been reported in various plant taxa, such as *AtMYB75* (*PAP1*), *AtMYB90* (*PAP2*), *AtMYB113*, and *AtMYB114* in *Arabidopsis* (Borevitz et al., 2000; Stracke et al., 2001; Zimmermann et al., 2004; Stracke et al., 2007; Gonzalez et al., 2008), *ROSEA1* and *ROSEA2* in *Antirrhinum* (Schwinn et al., 2006; Shang et al., 2011), *VvMYBA1* and *VvMYBA2* in *Vitis* grapevine (Walker et al., 2007), and *Ruby* in *Citrus* (Butelli et al., 2012; Huang et al., 2018).

The first flavonoid-related R2R3-MYB repressor, AmMYB308, was characterized from *Antirrhinum majus* (Tamagnone et al., 1998). R2R3-MYB repressors belong to the same R2R3-MYB family subgroup and are characterized by two conserved motifs in the C-terminal region, C1 (lsrGIDPxT/NHR) and C2/EAR (pdLNLD/EL) (Chen et al., 2019). They have also been identified in many plant taxa, such as *AtMYB4* in *Arabidopsis* (Hemm et al., 2001), PhMYB27 in petunia (Albert et al., 2014), and VvMYBC2-L1/3 and VvMYB4-like in *Vitis* (Cavallini et al., 2015; Pérez-Díaz et al., 2016). Many of these repressors have a function similar either to *Arabidopsis* AtMYB4, which represses the transcription of lignin and general phenylpropanoid genes, or to strawberry FaMYB1 (Aharoni et al., 2001), which negatively regulates transcription of anthocyanin and/or proanthocyanidins (PA) pathway genes (Jin et al., 2000; Cavallini et al., 2015; Jun et al., 2015; Liu et al., 2019). However, the mechanism by which these repressors regulate anthocyanin biosynthesis remains mostly unclear.

Alternative splicing (AS) is a common phenomenon in plants and leads to the production of more than one mRNA transcript from the same gene. AS not only contributes to proteome diversity but can also generate truncated proteins that are potentially regulatory or detrimental to cells (Syed et al., 2012). AS was found to occur in >60% of intron-containing genes in *Arabidopsis* (Marquez et al., 2012), and a similar percentage was estimated in other plant species (Syed et al., 2012). However, to date AS has only been detected in a limited number of R2R3-type MYB genes (Li et al., 2006), none of which have been identified as being involved in the anthocyanin biosynthesis pathway.

*Brassica napus* L. (rapeseed, oilseed rape, and canola) (AACC, 2n=38) is an important and high yielding global oil crop and, along with *Arabidopsis*, is a member of the Brassicaceae. It is thought to have originated by allopolyploidisation following spontaneous interspecific hybridization between the diploids, *B. rapa* (AA, 2n = 20) and *B. oleracea* (CC, 2n = 18), about 7,500 years ago (Chalhoub et al., 2014). Considerable variation in anthocyanin pigmentation is found in leaves, stems, and fruit of rapeseed as well as in various crop types of *B. oleracea* and *B. rapa* (Izzah et al., 2013). Several R2R3-MYB genes have been reported to regulate the expression of anthocyanin pathway structural genes in *B. rapa* (He et al., 2016) and *B. oleracea* (Yuan et al., 2009; Zhang et al., 2015). The distinct purple color formation found in some cultivars of cauliflower, cabbage, and kale/kohlrabi have been reported to be caused by the insertion of different transposons, point mutations, and/or a 1-bp insertion in the promoter region of the *BoMYB2* gene (Chiu et al., 2010; Chiu and Li, 2012; Yan et al., 2019). However, due to the triplicated genome origins of the *Brassica* lineage, multiple paralogs of these orthologs are usually present within the diploids *B. rapa* (A genome) and *B. oleracea* (C genome), leading to many more copies within the allotetraploid *B. napus* (AC genome). This complexity leads to uncertainty in establishing which functional alleles contribute to a purple phenotype in *B. napus*, with multiple paralog candidates on each of the A or C genomes.

In the present study, we identified a rapeseed R2R3-MYB activator, *BnaPAP2.A7* (BnaA07g25800D). Functional analysis indicated that *BnaPAP2.A7* acts as a positive regulator of anthocyanin accumulation in leaves. In addition, *BnaPAP.A7* produced three AS isoforms that are associated with variation in transcript abundance, protein sequence, and function. The wild-type spliced isoform encodes a protein with a complete R2, R3 repeat and C terminal domain, while the two AS isoforms produced truncated proteins lacking a partial R3 repeat and a complete C terminal domain. Interestingly, transgenic analysis indicated that the two spliced isoforms could significantly downregulate the expression of general phenylpropanoid genes as well as the expression of anthocyanin biosynthesis genes. We discuss the potential mechanism involving AS that is associated with the balance of positive and negative regulation of anthocyanin biosynthesis by the same R2R3-MYB protein.

## Materials and Methods

### Plant Materials and Phenotypic Anatomy

A *B. napus* alien introgression line was generated from the sequential crosses of (*B. rapa* ssp. *chinensis* L. *×* *Orychophragmus violaceus*) *×* *B. napus* by successive phenotypic selection and cytological observation (Li and Heneen, 1999; Xu et al., 2019). After selfing for more than 10 generations, this line possessed the same chromosome complement (2n=38) as *B. napus*, a normal meiotic behavior, and a good seed-set. It was characterized by purple pigmentation of all organs apart from yellow petals and named as PR (purple rapeseed). In addition, two homozygous accessions of *B. napus*, Zhongshuang 11 (ZS11) and Jia9709, which had a wild-type green phenotype were also used in this study for comparative transcriptome analysis and as a recipient for transformation, respectively. All plants were grown in the experimental field and greenhouse in Huazhong Agricultural University, Wuhan. To examine cells with purple pigmentation, young leaves were transversely sectioned by a free-hand section and examined with a Zeiss Axioscope photomicroscope equipped with a MRC digital camera. Transgenic *B. napus* plants were grown in an isolated experimental station. *Arabidopsis thaliana* and *Nicotiana benthamiana* plants were grown in a plant growth chamber at 20–22°C and 70% humidity under a photoperiod of 16-h light/8-h dark.

### Gene Cloning, Plasmid Construction, and Genetic Transformation

According to the reference from Darmor-*bzh*, a 3.66-kb genomic DNA fragment, including the 1.99-kb upstream regulatory sequence of the *BnaA07g25800D*, was amplified from PR using primer set PAP.A7-PF and PAP.A7-gR. PCR products were cloned into pMD™18-T (Takara, Dalian, China) and transformed into *Escherichia coli* (TransGen, Beijing, China) and sequenced. Because the translated amino acid level sequences of *BnaA07g25800D* have the highest similarity with *AtPAP2*, it was named *BnaPAP2.A7*. To obtain the coding sequence (CDS), the primer set PAP2.A7-CDS-PF and PAP2.A7-CDS-PR were used to amplify CDS from the young leaf cDNA of PR. Three fragments of different lengths were obtained and cloned into pMD™18-T, transformed into *E. coli* and sequenced. The 3.66-kb full-length gene, including 1.99-kb of the upstream regulatory sequence and the 1.67-kb coding region for *BnaPAP2.A7*, was amplified from the plasmid using primers PAP2.A7-KpnI-PF and PAP2.A7-BamHI-gR, and cloned into the KpnI-BamHI sites of binary vector pCAMBIA2301-nos. Subsequently, we amplified the 1.99-kb upstream regulatory sequence from the plasmid using primer set PAP2.A7-KpnI-PF and PAP2.A7-BamHI-PR and cloned this into the *KpnI*-*BamHI* cloning sites of the binary vector pCAMBIA2301-nos. Three transcripts of *BnaPAP2.A7* were also amplified again from the plasmid using primer set PAP2.A7- BamHI-PF and PAP2.A7-BstEII-PR and cloned into the BamHI - BstEII cloning sites of the vector pCAMBIA2301 behind the 1.99-kb upstream regulatory promoter, respectively. The four plasmids above were finally introduced into the *Agrobacterium tumefaciens* strain GV3101 (pMP90) and transformed into Jia9709 according to Cardoza and Stewart (2003).

### Subcellular Localization

Three transcript fragments were amplified and cloned into the *Pml*I-A*si*SI sites of pDOE20 (Gookin and Assmann, 2014) to generate mVenus-BnaPAP2. A7 (YFP- BnaPAP2. A7) fusion protein. The full-length cDNA fragments of *AtPAP2* from *Arabidopsis thaliana* were amplified and cloned into the *Bam*HI-*Xba*I sites of pDOE20 to generate the mTurquoise2-AtPAP2 (CFP-AtPAP2) fusion protein. All the resulting plasmids were introduced into *A. tumefaciens* EHA105, which were then infiltrated into 4-week-old *N. benthamiana* leaves for transient expression. In brief, the *A. tumefaciens* strains, containing the pDOE20 recombinant plasmid that expresses CFP-AtPAP2 and YFP-BnaPAP2. A7, also expresses the silencing suppressor p19 of *Tomato bushy stunt* virus. These were harvested and re-suspended to a final density of 0.2 at 600 nm (OD_600_) in an infiltration buffer (150 mM acetosyringone, 10 mM MgCl_2_ and 10 mM MES). The *Agrobacterium* suspension was injected into expanded leaves of 4-week-old tobacco plants. The tobacco leaves were imaged under a Zeiss LSM 700 confocal microscope after three days of infiltration. Excitation of YFP was performed using an argon laser line at 488 nm and a cyan fluorescent protein (CFP) with 405 nm (Qin et al., 2017).

### Yeast Two-Hybrid Assays

Yeast two-hybrid assays were conducted using the Frozen-EZ Yeast Transformation II Kit (ZYMO RESEARCH, USA, http://www.zymoresearch.com). The PCR products of *AtTT8* from *A. thaliana* were cloned into the pGBKT7 (BD) vectors, and three transcripts from PR leaves cDNA were independently cloned into the pGADT7 (AD) vectors. The PCR products of *AtPAP2* from *A. thaliana* were cloned into the pGADT7 (AD) vectors as a positive control. The transformation of AH109 cells was performed according to the protocol from Frozen-EZ Yeast Transformation II Kit. The transformed yeast cells were spread on the medium SD/-Leu/-Trp. For the yeast two-hybrid assay, some baits contained *AtTT8* plasmids while others did not. The preys used were with transcript or were empty AD vector plasmids introduced into yeast AH109 cells. The colony that was grown on the medium SD/-Leu/-Trp were chosen by selective medium SD/-Leu/-Trp/-His/-Ade to determine the interactions (Qin et al., 2017).

### RNA Preparation, Reverse-Transcription PCR, and qRT-PCR Analysis

The seventh leaf of PR, ZS11, and positive and negative transgenic T_2_ plants were successively collected with three biological repeats and immediately stored in liquid nitrogen for RNA extraction. Total RNA was extracted from various plant tissues using Eastep Super Total RNA Extract Kit (Promega, Shanghai, China) supplemented with RNase-free DNaseI to remove contaminated DNA according to the manufacturer’s instructions. First-strand cDNA was synthesized using a RevertAid First Strand cDNA Synthesis Kit (Thermo, USA, https://www.thermofisher.com/cn/zh/home.html). The cDNA was amplified on a CFX96TM Real-time PCR Detection System (Bio-Rad, Germany, http://www.bio-rad.com/). Eight genes *PAL1* (BnaA04g21230D), *C4H* (BnaA04g21230D), *CHS* (BnaA10g19670D), *F3’H* (BnaA10g23330D), *MYB4* (BnaC03g60080D), *ANS* (BnaA01g12530D), *DFR* (BnaC09g17150D), and *MYBL2* (BnaA02g15320D) were selected for qRT-PCR confirmation of the expression level revealed by RNA-seq analysis. The specific quantitative primers for different genes and their transcripts were designed using Primer 5.0. qRT-PCR assays with three biological replicates and three technical repetitions were performed using a Luna Universal qPCR Master Mix (Biolabs, USA) on a Bio-Rad CFX96 Real-Time Detection System (Bio-Rad, Germany, http://www.bio-rad.com/). The *Bnaactin3* gene was used as an internal control for data normalization, and quantitative variation in the different replicates was calculated using the delta-delta threshold cycle relative quantification method as described previously (Fu et al., 2018).

### RNA-Seq and Gene Ontology (GO) Enrichment Analysis

We first re-analyzed the leaf transcriptomic data of PR and ZS11 (Mushtaq et al., 2016) young leaf RNA-seq data (each with two duplicates). In order to investigate the potential gene expression changes in transgenic lines, we also performed RNA-seq analysis. Total RNA of transgenic T_2_ lines (three biological duplicates each from independent transformant) containing full-length *BnaPAP2. A7*, *BnaPAP2. A7-395*, and *BnaPAP2. A7-910* as well as negative transgenic T_2_ plants (three biological duplicates) were used for the sequencing library construction. The RNA-seq libraries were constructed according to the user manual (Illumina, http://www.illumina.com/) and sequenced to produce 150-bp paired-end reads. Low-quality reads were removed from the raw reads using the Cutadapt (Martin, 2011) and Trimmomatic (Bolger et al., 2014) software. Clean reads were mapped to the *B. napus* Darmor-*bzh* genome sequence (http://www.genoscope.cns.fr/brassicanapus/) (Chalhoub et al., 2014) using TopHat2 (Kim et al., 2013). The read counts of each gene were calculated using the HTseq-count function in the HTseq software package (Anders et al., 2015). The R program DEseq2 (http://www.bioconductor.org/packages/release/bioc/html/DESeq2.html) was used to identify differentially expressed genes (DEG)s between purple and green plants based on the criteria: padj < 0.01 & log2FoldChange > 2. Gene expression levels were calculated in terms of Fragments Per Kilobase of exon model per Million mapped reads (FPKM) using Cufflinks (Trapnell et al., 2012). The GO enrichment analysis was performed for DEGs using TBtools software (Chen et al., 2018). The top 20 enriched gene ontology (GO) terms for the comparative transcriptome DEGs of full-length gene, *BnaPAP2.A7-395*, and *BnaPAP2.A7-910*-positive transgenic plants compared with Jia9709 plants were used to produce the GO enrich diagram.

### Sample Preparation, Anthocyanin Content Determination, and Metabolic Analysis

Young leaves from three biological replicates of PR, Jia9709, and the positive transgenic lines were collected. For the full-length *BnaPAP2.A7*, *BnaPAP2.A7-395*, and *BnaPAP2.A7-910-*positive lines, three T_2_ lines were chosen, and three plants of each were collected as nine biological replicates. All samples were immediately stored in liquid nitrogen for later anthocyanin extraction. A total of 0.5 g of frozen powder was placed into a 5-mL screw-cap tube with 5mL of methanol/water/acetic acid (85:15:0.5; v, MeOH/H2O/AcAc). The sample was then put on ice in the dark with sonication for 30 min and shaken at 4°C in the dark overnight. The supernatant was collected after centrifuging at 12,000 x g for 10 min and was further diluted and filtered twice using a 0.22 µm PTFE filter (Cameo 25F, Micron Separations Inc., Westboro, MA) before injection for metabolomics analysis (Fu et al., 2018).

For the anthocyanin content analysis, firstly, a dilution series of standard cyanidin (C_15_H_11_ClO_6_; Solarbio, Beijing, China) was measured at 520 nm using a spectrophotometer to obtain a standard curve of anthocyanin content and absorbance. Then, the absorbance of the treated sample was obtained using a spectrophotometer, and the anthocyanin content was calculated from the obtained standard curve.

Metabolomics analysis was performed on a Waters Acquity UPLC system connected to a Synapt G2-XS QToF mass spectrometer, which was equipped with an electrospray ionization source (Waters, Milford, MA) according to methods previously described (Fu et al., 2018). However, we only collected anthocyanin related metabolites, which have an absorption peak at 530 nm. Data processing was performed using the MarkerLynx application manager for Masslynx4.1 software. Principal component analysis (PCA) and cluster analysis of anthocyanin content based on anthocyanin type and content from different plants was performed with MetaboAnalyst (https://www.metaboanalyst.ca/MetaboAnalyst).

### Phylogenetic Analysis

Protein sequences of PAP1, PAP2, MYB113, and MYB114 from *Arabidopsis thaliana* and homologs in *B. rapa*, *B. oleracea*, and *B. oleracea* were aligned using MEGA7 (Kumar et al., 2016), as were evolutionary analyses. The evolutionary history was inferred by using the Maximum Likelihood method based on the JTT matrix-based model (Jones et al., 1992). The tree with the highest log likelihood (-874.7994) is shown. Initial tree(s) for the heuristic search were obtained automatically by applying Neighbor-Join and BioNJ algorithms to a matrix of pairwise distances estimated using a JTT model, and then selecting the topology with superior log likelihood value. The tree is drawn to scale, with branch lengths proportional to the number of substitutions per site. The analysis involved 19 amino acid sequences. All positions containing gaps and missing data were eliminated. There were a total of 130 positions in the final dataset.

## Results

### Characterization of Sequence Variation and Expression of *BnPAP2.A7* in Purple and Green Rapeseed

**Figure 1:**
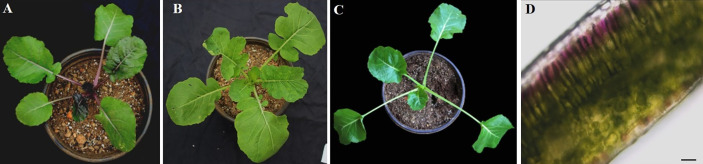
Characterization of anthocyanin accumulation in leaves of purple rapeseed. **(A–C)** Different phenotype of young plant of PR **(A)** and ZS11 **(B)** planted outdoor and PR in the glass greenhouse **(C)**. **(D)** Hand section of a young leaf of PR. Scale bar: 10μm.

**Figure 2:**
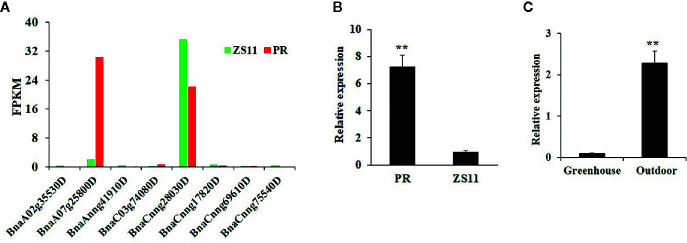
Characterization of the expression of *BnPAP2.A7* in purple and green rapeseed. **(A)** The fragments per kilobase of exon model per million mapped reads (FPKM) of eight *AtPAP2* homologous genes in leaves of PR and ZS11. **(B)** qRT-PCR analysis of *BnaPAP2.A7* in leaves of PR and ZS11 planted outside. **(C)** qRT-PCR analysis of *BnaPAP2.A7* in leaves of PR planted outside and in glass greenhouse. **P < 0.01, one-way ANOVA test. Error bars represent the SD of three replicates.

At the seedling stage, purple rapeseed (PR) displays distinctive purple leaves and petioles (**Figure 1A**) compared to the green rapeseed ZS11 (**Figure 1B**). In addition, the purple phenotype of PR is not visible when planted in a glass greenhouse (**Figure 1C**). This result suggests that ultraviolet radiation, which is largely blocked by glass, may be required for purple color formation. As shown in leaf sections, the purple pigments mainly accumulated in the epidermal cells (**Figure 1D**). Comparative leaf transcriptome analysis revealed significant upregulation of homologous late-biosynthesis genes (LBGs) involved in the anthocyanin biosynthesis pathway, including *DFR*, *ANS*, and UGFT, in PR compared with ZS11 (Mushtaq et al., 2016). These results suggested that allelic variation of one of the MYB-bHLH-WD40 (MBW) components may result in the activation of LBGs and lead to the purple color formation of PR. We therefore identified all homologous genes, classified as *Arabidopsis*, as MBW components within the reference *B. napus* genome (Darmor-*bzh*) (**Table S1**) and discovered that two of the eight homologous genes of *AtPAP2*, *BnaCnng28030D* and *BnaA07g25800D*, were differentially expressed between PR and ZS11. However, only *BnaA07g25800D* was highly expressed in PR, which is consistent with its purple-color phenotype (**Figure 2A**). Quantitative RT-PCR (qRT-PCR) analysis further confirmed that *BnaA07g25800D* was expressed at a significantly higher level in the leaves of PR than in those of ZS11 (**Figure 2B**). We also found that *BnaA07g25800D* was mostly absent in the leaves of greenhouse grown plants but was highly expressed in the leaves of plants grown outdoors (**Figure 2C**). BnaA07g25800D was thus considered the key transcription factor involved in the regulation of anthocyanin biosynthesis in leaves of PR.

**Figure 3:**
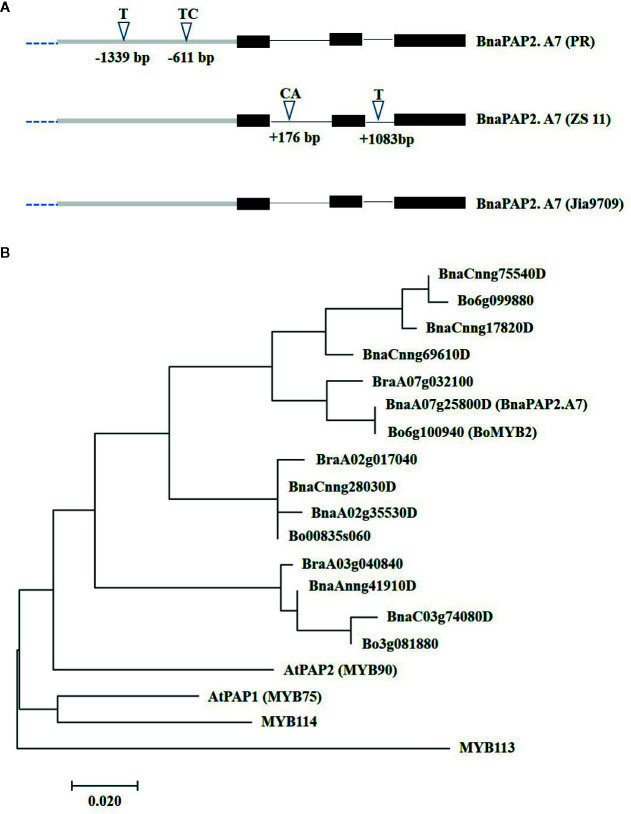
Allelic variation and phylogenetic analysis of *BnaPAP2. A7.* **(A)** Allelic variation of *BnaPAP2. A7* including 1.99 kb promoter region and 1.67 kb coding region between PR and ZS11. **(B)** Phylogenetic tree derived from amino acid sequences of R2R3-MYBs in *B. napus*, *B. rapa*, and *B. oleracea* showed homology to *Arabidopsis* *AtPAP2*.

To determine the full-length sequences of the corresponding alleles from PR, ZS11, and J9709, we isolated the gene using overlapping primers that bound specifically to the genomic region of *BnaA07g25800D*. J9709 also has a green phenotype and is particularly susceptible to genetic transformation. The PCR products represented about 3.66 kb of genomic fragments, comprised of a 1.67 kb transcribed region and a 1.99 kb promoter region. Compared with ZS11, the PR allele included a 1bp insertion (T) at -1,339 and a 2bp insertion (TC) at -611bp within the promoter region (**Figure 3A**). In the transcribed region, there is a 2 bp (CA) insertion in ZS11 at +176 in intron 1, and a 1bp (T) insertion in intron 2 at +1,083 (**Figure 3A**). The sequence of the J9709 allele is identical to ZS11 in both the promoter and transcribed regions and is identical to the PR allele within the introns (**Figure 3A**). The translated amino acid sequences of the three alleles were identical. As the translated amino acid sequences of *BnaA07g25800D* were most similar to those of AtPAP2, it was named *BnaPAP2.A7*. Phylogenetic analysis revealed that *BnaPAP2.A7*, *BraA07g032100*, and *Bo6g100940* (*BoMYB2*) are classified within the same subgroup (**Figure 3B**). This suggests that these genes may have similar functions.

### *BnaPAP2.A7* Produces Three Transcript Isoforms *via* AS

**Figure 4:**
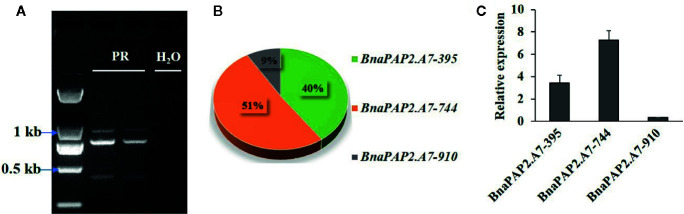
Characterization the expression of three alternative spliced transcripts produced by *BnaPAP2. A7*. **(A)** Detection of three isoforms by agarose gel electrophoresis. **(B)** Expression abundance analysis of three isoforms by colony PCR and agarose gel electrophoresis. **(C)** qRT-PCR analysis of three isoforms.

**Figure 5:**
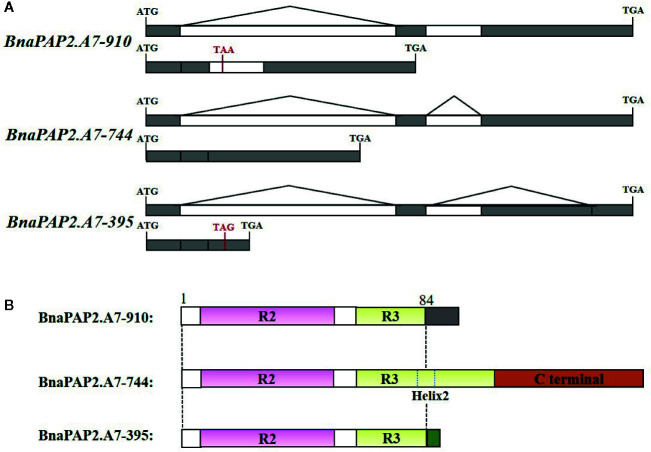
Production, gene structure, and proteins sequence of three isoforms of *BnaPAP2. A7*.*PR.* **(A)** Production and gene structure of the three isoforms. **(B)** Schematic diagram showing the protein sequence encoded by three isoforms. Gray and green boxes represent the new short C-terminal sequence that are completely different from those of original C-terminal.

Interestingly, when we isolated the full-length cDNA of *BnaPAP2.A7* from PR we found three alternative splicing (AS) isoforms of 910 bp, 395 bp, and 744 bp (**Figure 4A**). To analyze their relative expression, RT-PCR products amplified by primers for coding regions were randomly cloned and detected by colony PCR and agarose gel electrophoresis. Based on 900 clones, the results indicated transcript abundance decreasing from isoform 744 (51.1%) > isoform 395 (40.1%) > isoform 910 (8.8%) (**Figure 4B**). qRT-PCR analysis using specific primer pairs for each isoform also revealed a similar expression trend (**Figure 4C**). When these isoforms were compared with the predicted coding DNA sequence (CDS) of BnaA07g25800D, the 744 isoform with three exons was determined to be the normal transcript. However, 910 contained these three exons and an additional 166 bp of the second intron, whilst 395 contained the first two exons and the first 349 bp of the third exon (**Figure 5A**). At the amino acid level, the 744 isoform-translated proteins have 244 amino acids and the complete R2, R3, and C-terminal domains. However, due to AS, the 910 and 395 isoforms contain premature stop codons and produce proteins composed of 103 and 108 amino acids, respectively (**Figure 5A**). The latter two proteins thus contain a complete R2 repeat but lack both a section of R3 from the middle region of the second α-helix as well as the complete original C-terminal domain (**Figure 5B**).

### Truncated Proteins Encoded by Two Spliced Isoforms Are Localized in the Nucleus but Have Lost the Ability to Interact With the bHLH Protein

**Figure 6:**
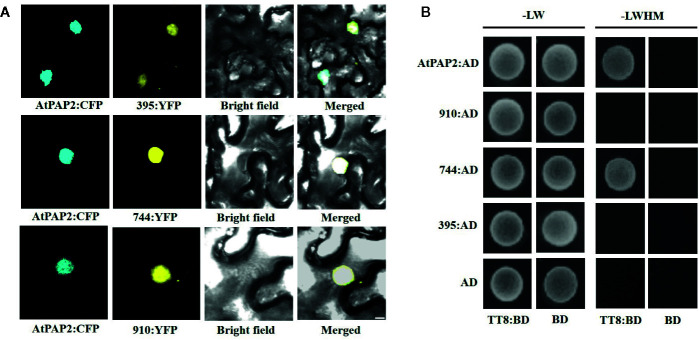
Subcellular localization of proteins encoded by three isoforms and their ability to bind to bHLH protein revealed by Yeast two-hybrid analyses. **(A)** BiFC interaction assays of BnaPAP2.A7-395, BnaPAP2.A7-744, and BnaPAP2.A7-910 with AtPAP2. Partial YFP fusion constructs were expressed transiently in *N. benthamiana* leaves. AtPAP2-CFP was used as a known nuclear marker. Scale bar: 100 μm. **(B)** Yeast two-hybrid analyses of interactions between three transcripts and AtTT8. 395: BnaPAP2.A7-395; 744: BnaPAP2.A7-744; 910: BnaPAP2.A7-910. LW: Leucine and Tryptophan; LWHM: Leucine, Tryptophan, Histidine and Methionine.

To explore the subcellular localization of the proteins encoded by the three transcripts, a pDOE20 plasmid co-expressing BnaPAP2.A7-yellow fluorescent protein (YFP) and AtPAP2-cyan fluorescent protein (CFP) fusion proteins were introduced into tobacco leaves for transient expression analysis. The results demonstrated that the proteins produced by the three isoforms of *BnaPAP2.A7* co-localized with AtPAP2 in the nucleus (**Figure 6A**). We wished to investigate whether the loss of the R3 and C-terminal domain affects the affinity between BnaPAP2.A7 and bHLH, which is important in subsequent MBW complex formation. We therefore cloned *AtTT8* and performed a yeast two-hybrid assay of the interaction between AtTT8 and proteins encoded by the three isoforms, with AtPAP2 used as a positive control. AtTT8 strongly interacted with the 744-encoded protein in yeast cells, but no binding was detected between AtTT8 and proteins produced by 910 or 395 (**Figure 6B**). We therefore speculate that only the 744 isoform-encoded protein participates in the formation of MBW complexes and regulates anthocyanin biosynthesis.

### Transgenic Analysis of Full-Length *BnaPAP2.A7* and Three AS Transcripts in Green Rapeseed

**Figure 7:**
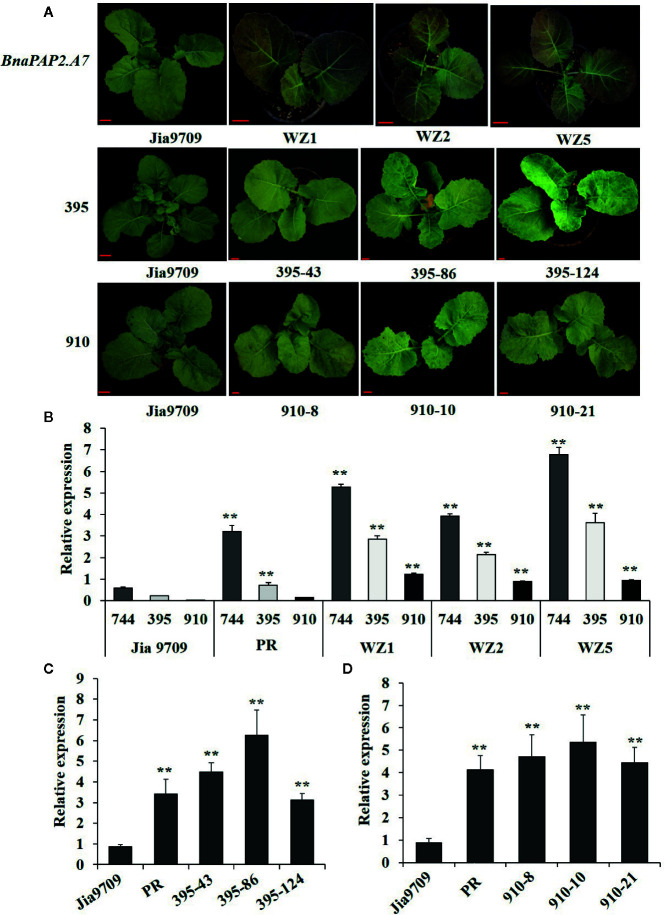
Transgenic analysis of full-length *BnPAP2.A7* and three isoforms in green rapeseed. **(A)** Phenotypes of transgenic lines at the seedling stage containing full-length PR *BnaPAP2. A7*, 395, and 910 isoform driven by the same 1.99kb promoter of *BnaPAP2. A7*. Scale bar: 1 cm. **(B)** qRT-PCR analysis of three isoforms in transgenic plants with full-length *BnaPAP2. A7*. **(C**, **D)** qRT-PCR analysis of 395 **(C)** and 910 **(D)** isoforms in respective transgenic plants. **P < 0.01, one-way ANOVA test. Error bars represent the SD of three replicates.

To test our speculation further, we constructed pCAMBIA2301-based expression vectors containing full-length genomic fragments of *BnaPAP2.A7.PR* that included both the 1.99 kb promoter and 1.67 kb transcribed regions as well as the three isoforms driven by the same 1.99 kb promoter, and we transformed these into the green-leaved Jia9709. This gave rise to 15 positively transformed plants harboring the full-length sequence of *BnaPAP2.A7.PR*, 56 for the 395 isoform, and 33 for the 910 isoform. However, no transformed plant harboring the 744 isoform was obtained, despite attempts using various infection conditions (**Table S2**) and different expression vectors. Of the 15 positive plants carrying the full-length *BnaPAP2.A7.PR* gene, six displayed purple leaves in the T_0_ generation, three of which (WZ1, WZ2, and WZ5) were used for further study. A deeper purple color was observed in the subsequent T_1_ (data not shown) and T_2_ generations of all three lines (**Figure 7A**). However, no purple color was observed in the leaves or other organs of transgenic plants harboring the 910 or 395 isoforms in the T_1_ (data not shown) and T_2_ generations (**Figure 7A**). RT-PCR analysis confirmed that Jia9709 carrying full-length *BnaPAP2.A7.PR* also produced three isoforms, each having a significantly higher transcript level in the T_2_ transgenic plants than those in control plants (**Figure 7B**). Meanwhile, the 910 and 395 isoforms also had a significantly higher transcript level in transgenic plants harboring the 910 and 395 isoforms, respectively (**Figures 7C, D**).

In the T_2_ generation, the WZ1, WZ2, and WZ5 lines had an average anthocyanin content of 3.13~4.1 mg/g fresh weight—significantly higher than that presented by Jia9709 (0.61~1 mg/g). However, the leaf anthocyanin content of the 910 (0.66~0.85 mg/g) and 395 (66~0.89 mg/g) lines were similar to the control line (**Figure S1A**). Non-target metabolic analysis identified similar numbers of anthocyanin related metabolites in the control (921 metabolites) as well as in the full-length *BnaPAP2.A7.PR* (939), 910 isoform (912), and 395 isoform (905) transgenic lines (**Supplementary datasheet 1**). However, metabolite-based PCA analysis revealed that the three transgenic lines and controls each formed distinct separated groups of plants (**Figure S1B**). Cluster analysis shows that while plants of the 395 and 910 transgenic lines had similar metabolite profiles, each were significantly different from those of the *BnaPAP2.A7.PR* full-length transgenic lines and controls (**Figure S1C**). Taken together, these results indicated that the proteins encoded by 910 and 395 failed to promote anthocyanin synthesis. Therefore, it is reasonable to consider that the 744 isoform-encoded proteins may participate in the formation of MBW complexes and regulate anthocyanin biosynthesis, although no plants transformed with this isoform were obtained.

### The Potential Negative Regulation of Anthocyanin Biosynthesis by Two Spliced Isoforms

**Figure 8:**
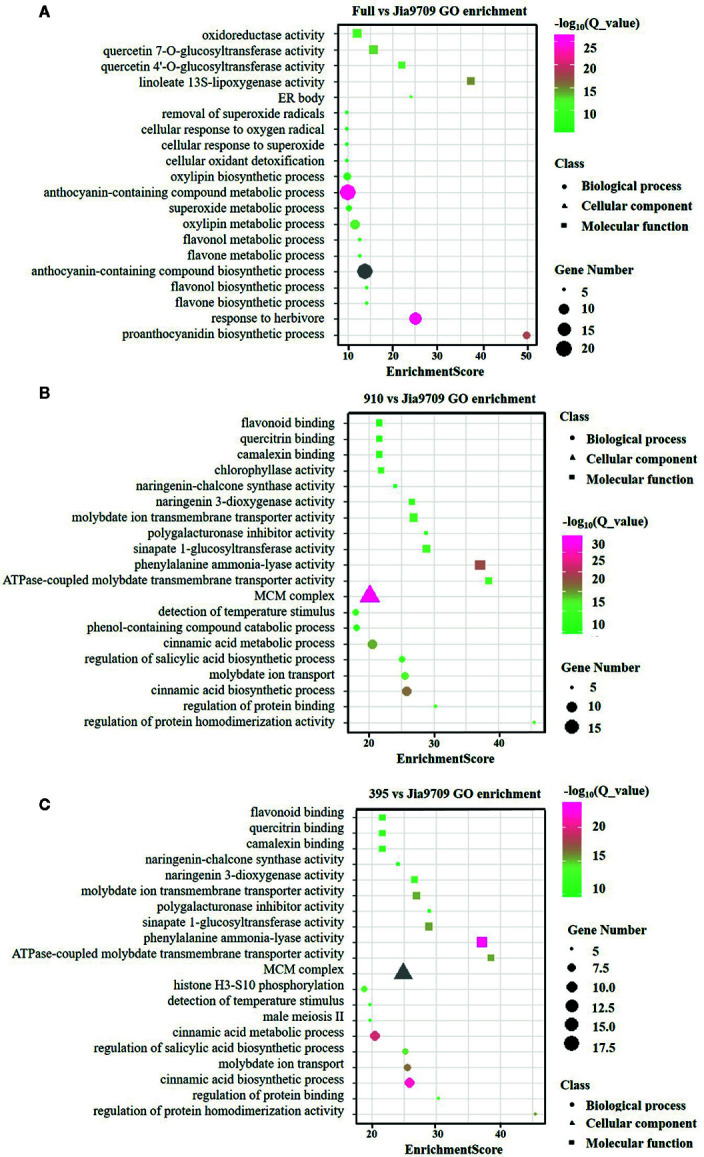
GO enrichment analysis of the DEGs in transgenic lines. **(A-C)** Top twenty enriched GO categories of DEGs in full-length *BnPAP2.A7* transgenic lines **(A)**, 395 **(B)**, and 910 **(C)** isoform transgenic lines.

In order to analyze the potential function of the 395 and 910 isoforms, we performed RNA-seq analysis of full-length, 395 and, 910 transgenic plants as well as plants subjected to transformation but with no inserts, which served as controls. Compared to the control plants this identified 391 (374 up/17 down) differentially expressed genes (DEGs) in transgenic plants harboring the full-length gene, 3,820 (1,348 up/2,472 down) in the 395 isoform and 3,598 (1,238 up/2,360 down) in the 910 isoform (**Supplementary datasheet 2**). Gene ontology (GO) enrichment analysis revealed a substantial difference between the DEGs of the three types of transgenic plants (**Figures 8A–C**). DEGs of full-length *BnaPAP2. A7.PR* transgenic plants were enriched in several GO categories involved in flavonoid, anthocyanin, and proanthocyanidin biosynthetic and metabolic processes. However, DEGs in the 395 and the 910 transgenic plants were enriched in GO categories mainly involved in the phenol-containing compound catabolic process, the cinnamic acid biosynthetic and metabolic process, and other molecular function such as flavonoid binding (**Figures 8A–C**). In particular, all of the 16 DEGs involved in anthocyanin biosynthesis showed significantly higher transcript abundance in full-length *BnaPAP2. A7.PR* transgenic plants. These included F3’H, FLS, DFR, and UGFT as well as the anthocyanin transporter TT19 and transcription factors such as *PAP2* and *TT8* (**Supplementary datasheet 3**; **Figure S2A**). In contrast, most of the DEGs involved in anthocyanin biosynthesis in the 395 (45/57) and 910 (33/39) transgenic lines were significantly downregulated, including *PAL*, *C4H*, and *4CL* and almost all EBGs (**Supplementary datasheet 3**, **Figures S2B, C**). Additionally, transcription factors such as MYB111 and MYB12 were significantly downregulated while MYBL2 was significantly upregulated (**Supplementary datasheet 3**). These results suggest that the two alternatively spliced transcripts disturbed the pattern of genome-wide gene transcription and repressed the expression of many anthocyanin related genes.

**Figure 9:**
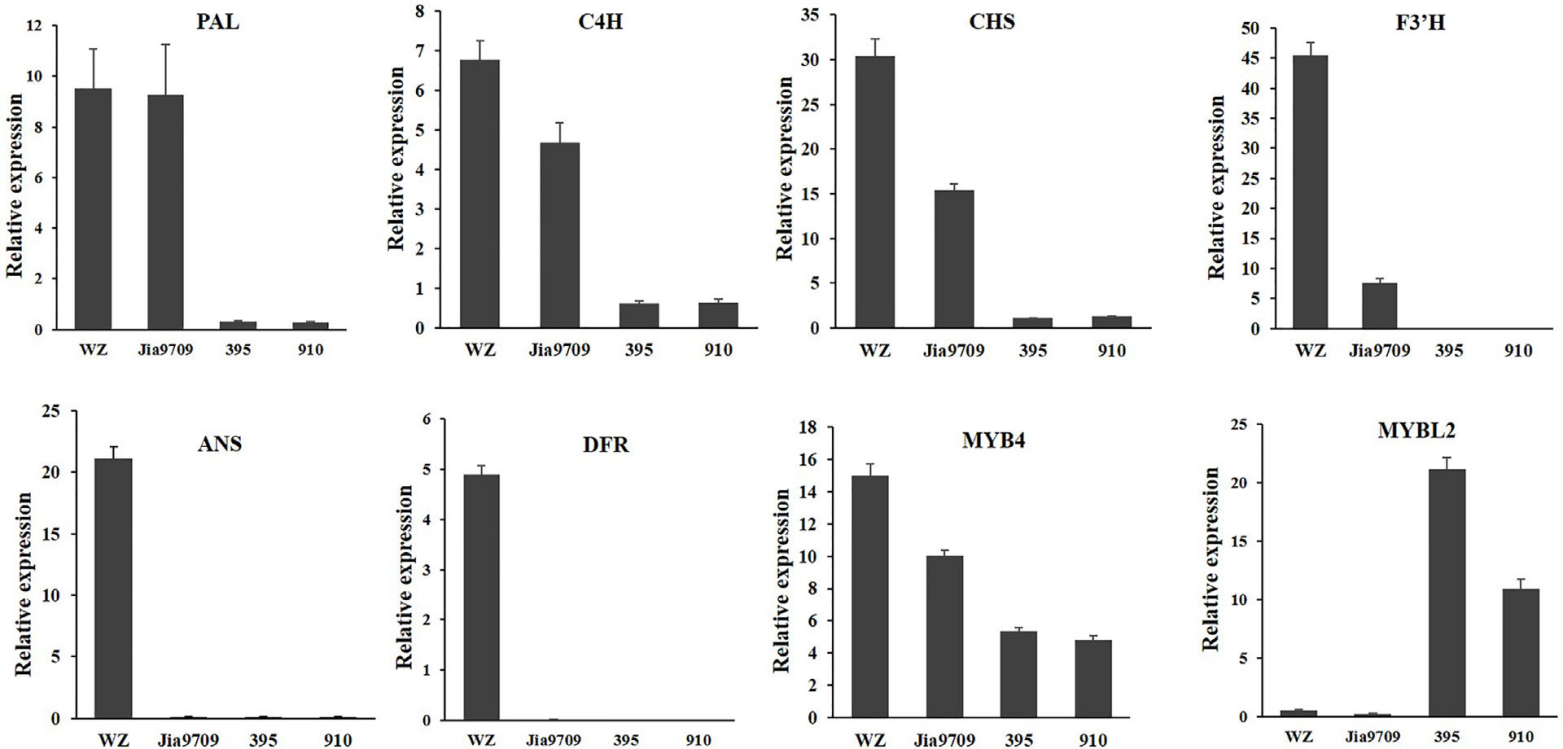
qRT-PCR analysis of eight anthocyanin biosynthesis genes in J9709, transgenic plants with full-length *BnaPAP2. A7* (wz), 395 and 910 isoforms.

In order to confirm the transcriptional pattern revealed by the RNA-seq analysis, we performed qRT-PCR analysis for eight genes, including two phenylpropanoid pathway genes, two EBGs, two LBGs, as well as two transcription factors (**Figure 9**). All genes showed a similar relative expression level in transgenic lines and J9709 as revealed by RNA-seq analysis. The expression of *PAL1* (BnaA04g21230D) in full-length transgenic lines was similar as in the J9709 control, but it was significantly downregulated in the 395 and 910-isoform transgenic lines. *C4H* (BnaA04g21230D), *CHS* (BnaA10g19670D), *F3’H* (BnaA10g23330D), and *MYB4* (BnaC03g60080D) were significantly upregulated in full-length transgenic lines but were significantly downregulated in the 395 and 910 transgenic lines. *ANS* (BnaA01g12530D) and *DFR* (BnaC09g17150D) were significantly upregulated in full-length transgenic lines but were almost silenced in J9709 as well as in the 395 and 910 transgenic lines. Interestingly, *MYBL2* (BnaA02g15320D) was expressed at a very low level both in J9709 as well as in full-length transgenic lines but was significantly upregulated in the 395 and 910-isoform transgenic lines (**Figure 9**).

## Discussion

In this work, we have demonstrated that one of the eight homologs of *AtPAP2* in rapeseed, *BnaPAP2.A7*, is a key gene responsible for purple leaf formation. Phylogenetic analysis indicated that the A genome *BnaPAP2.A7* is an orthologous gene of the C genome *BoMYB2*, which has previously been shown to be responsible for purple traits in cauliflower, kale, kohlrabi, and cabbage (Chiu et al., 2010; Yan et al., 2019). Moreover, BoMYB2 has been shown to be involved in MYB–bHLH–WD40 formation by direct interaction with BobHLH1. The expression of *BoMYB2* in *Arabidopsis* significantly upregulates a subset of anthocyanin structural genes, including *F3’H*, *DFR*, and *ANS* (Chiu and Li, 2012). Here, both the RNA-seq based transcript and qRT-PCR analysis indicated a significantly higher expression level of *BnaPAP2.A7* in purple leaves of PR than that in green leaves of ZS11 (**Figure 2**). Transgenic analysis in J9709 of the full-length *BnaPAP2.A7.PR* allele that included the 1.99 kb promoter region further confirmed elevated expression in leaves; significant upregulation of late anthocyanin biosynthesis genes appears to contribute to anthocyanin accumulation (**Figure 7**). Yeast two-hybrid studies also indicated that BnaPAP2.A7 could interact with AtTT8 *in vitro* (**Figure 6**). As found with BoMYB2, in leaves of *B. napus*, BnaPAP2.A7 appears to be involved in the formation of MYB–bHLH–WD40 complexes, affecting transcriptional regulation of late anthocyanin biosynthesis genes. However, it should be noted that only transgenic plants with *BnaPAP2.A7.PR* had significant purple color in leaves compared to the presence of purple color in the leaves, stem, and silique of PR. These results suggest that tissue-specific accumulation of anthocyanins in *B. napus* may be controlled by multiple copies of R2R3-MYB. Consistent with this, a paralog of *BnaPAP2.A7* on chromosome C6 has been shown to be responsible for the purple stem trait of PR, based on Bulked Segregant Analysis (BSA) mapping and cloning (Chen DZ et al., unpublished data).

Transgenic analysis indicated that the 1.99 kb promoter region from PR is capable of activating the expression of *BnaPAP2.A7* in different genetic backgrounds. However, only a single bp insertion (T) and 2bp insertion (TC) were present in the promoter region of *BnaPAP2.A7.* *PR* compared to ZS11 (**Figures 3** and **7**). In addition, *BnaPAP.A7* was highly expressed in both PR and transgenic J9709 in outdoor compared to greenhouse environments, which suggests that its expression may be UV dependent (**Figure 2**). However, the mechanism for such activation remains unknown since no known motif was identified at these sites. Similarly, the activation of the *BoMYB2* in purple cabbage was recently revealed to be caused by a point mutation at -408 and/or the 1-bp insertion at -95 in the promoter region (Yan et al., 2019). However, this was caused by the insertion of 4 kb Harbinger DNA transposon in purple cauliflower and by the insertion of a 7.6kb CACTA DNA transposon in both purple kale and kohlrabi (Chiu et al., 2010; Yan et al., 2019). In fact, the activation of the R2R3-MYB related to the anthocyanin biosynthesis is often caused by insertion of a transposon, as found for example in *Antirrhinum majus* (Lister et al., 1993), blood orange (Butelli et al., 2012), tea (Sun et al., 2016), and *Phalaenopsis* orchids (Hsu et al., 2019). Whether there are other possible elements that could enhance the expression of *BnaPAP2.A7* in PR beyond the 2 kb upstream is worthy of further study.

In this study, we found that two of the three alternative transcripts of *BnaPAP.A7* encoded truncated proteins lacking a partial R3 repeat and complete C terminal domain. Plant R2R3-MYBs contain two repeats (R2 and R3), each forming a helix-turn-helix motif. It has been proposed that the third helices of both the R2 and R3 domains are critical for the DNA-binding activity (Gabrielsen et al., 1991; Jia et al., 2004), whereas the first two α-helices of the R3 domain are involved in interactions with the bHLH protein (Zimmermann et al., 2004). Here, the two truncated proteins had both lost part of the second and third helices of the R3 repeat (**Figure 5**). Although there is evidence that the two truncated proteins localized in the nucleus, they had lost the ability to bind AtTT8 (**Figure 6**). Unexpectedly, the two proteins appeared to contribute to strong repression of genes involved in the phenylpopanid pathway and anthocyanin biosynthesis (**Figure S2**). This suggests that the two isoforms encoded by spliced transcripts act as repressors of anthocyanin biosynthesis, in direct contrast to the activator role encoded by the normally spliced isoform. Similarly, different alleles of Ruby2, a novel R2R3-MYB regulatory gene, have recently been shown to have opposite effects on the regulation of anthocyanin biosynthesis in *Citrus* (Huang et al., 2018). While *Ruby2^Full^* encodes an anthocyanin activator, *Ruby2^Short^* encodes a repressor with partial R2 and R3 repeats due to an alternative downstream ATG start codon and a 54 bp in-frame deletion. *Ruby2^Short^* has lost the ability to activate anthocyanin biosynthesis genes but retains the ability to interact with bHLH1, thus acting as a passive competitor in the regulatory complex (Huang et al., 2018). However, because the two rapeseed isoforms are unable to interact with AtTT8, it is unclear how they inhibit the expression of genes related to anthocyanin biosynthesis.

A possible explanation may be found by considering that many anthocyanin biosynthesis genes were significantly downregulated in the two AS transcript transgenic lines, which likely resulted from the repression of AtMYB4-like repressors (Chen et al., 2019). AtMYB4, the first transcription factor found to function as a repressor of the phenylpropanoid pathway, is light-responsive and affects one or more phenylpropanoid structural genes, especially for the C4H (Borevitz et al., 2000; Jin et al., 2000; Hemm et al., 2001). In *B. rapa* subsp*. rapa* cv. Tsuda, BrMYB4, a homologous gene of AtMYB4, has also been shown to bind to the promoter region of BrC4H directly and repress expression of C4H, with negative regulation of all anthocyanin biosynthesis. In addition, suppression of *ANS*, *DFR*, and UFGT has also been consistently observed in all transgenic tobacco and other plants by heterologous expression of AtMYB4-like repressors (Pérez-Díaz et al., 2016; Xu et al., 2017; Anwar et al., 2018). However, RNA-seq and qRT-PCR analysis did not detect activation of MYB4 homologous genes by the 395 and 910 isoforms. This indicates that, although the 395 and 910 isoforms repressed the anthocyanin biosynthesis genes, this was not by activating MYB4. Interestingly, an R3 MYB repressor, MYBL2, was upregulated in the 395 and 910 transgenic plants (**Supplementary Datasheet 3**; **Figure 9**). In *Arabidopsis*, AtMYBL2, which contains an R3 domain and part of an R2 domain, is also a negative regulator of anthocyanin biosynthesis (Dubos et al., 2008; Matsui et al., 2008). Phylogenetic analyses indicate that MYBL2-like R3-MYB repressors are closely related to R2R3-MYB repressors and might have originated *via* tandem duplication followed by large deletions in the R2 domain (Chen et al., 2019). It is possible that 395 and 910 isoforms could activate MYBL2 and so repress the expression of anthocyanin biosynthesis genes. A further study should be performed to test the overexpression of 395 and 910 isoforms in PR or other model plants such as tobacco to confirm their negative roles in anthocyanin biosynthesis. Further study should also focus on the potential binding activity of the two rapeseed truncated proteins using methods such as ChiP-seq.

In summary, our study has identified and characterized *BnaPAP2.A7*, an R2R3-MYB activator of anthocyanin biosynthesis, in *B. napus*. We have shown that it plays a key role in the regulation of purple color formation in leaves. We have also uncovered a potential new negative feedback loop controlling flavonoid biosynthesis involving alternatively spliced transcripts, which will contribute to our understanding of the homeostatic mechanism of flavonoid accumulation in plants.

## Data Availability Statement

Genomic sequences of BnaPAP2.A7 from PR, ZS11 and J9709 were deposited in NCBI, with accession number 2292245, 2292253, and MT438412. The coding sequence of two alternative spliced isoforms were deposited in NCBI, with accession number 2244186 and 2244486. Transcriptome data were deposited in NCBI under BioProjiect ID: PRJNA554517. The sequences of primer sets in this study were listed in **Table S3**.

## Author Contributions

XG, JW, and ZL conceived and designed the experiments. DC, YL, SY, QJ, and JQ performed the experiments. DC, XG, and JW analyzed the data, DC, XG, GK, and YL wrote the manuscript. All authors contributed to the article and approved the submitted version.

## Funding

This work was mainly supported by the National Key Research and Development Program of China (2016YFD0100202) and the Fundamental Research Funds for the Central Universities (2662018PY074). We thank Dr. Hongbo Liu for his help in metabonomics analysis and Mr. Qinghua Zhang for his help in RNA-seq analysis.

## Conflict of Interest

The authors declare that the research was conducted in the absence of any commercial or financial relationships that could be construed as a potential conflict of interest.
