# Supplementary material for: Alternatively Spliced BnaPAP2.A7 Isoforms Play Opposing Roles in Anthocyanin Biosynthesis of Brassica napus L
Source: Front Plant Sci. 2020 Aug 19;11:983. doi: 10.3389/fpls.2020.00983 (PMC7466728; doi:10.3389/fpls.2020.00983)
Supplement: Supplementary file 1 [file Table_1.docx]

**Alternative Splicing of *BnaPAP2.A7* Isoforms Play Opposing Roles in Anthocyanin Biosynthesis of *Brassica napus* L.**

Daozong Chen^1^, Yi Liu^1^, Qingdong Jin^1^, Jie Qiu^1^, Graham J King^2^, Jing Wang^1^, Xianhong Ge^1^*, Zaiyun Li^1^

^1^National Key Laboratory of Crop Genetic Improvement, National Center of Oil Crop Improvement (Wuhan), College of Plant Science and Technology, Huazhong Agricultural University, Wuhan 430070, People’s Republic of China

^2^Southern Cross Plant Science, Southern Cross University, Lismore, NSW 2480, Australia.


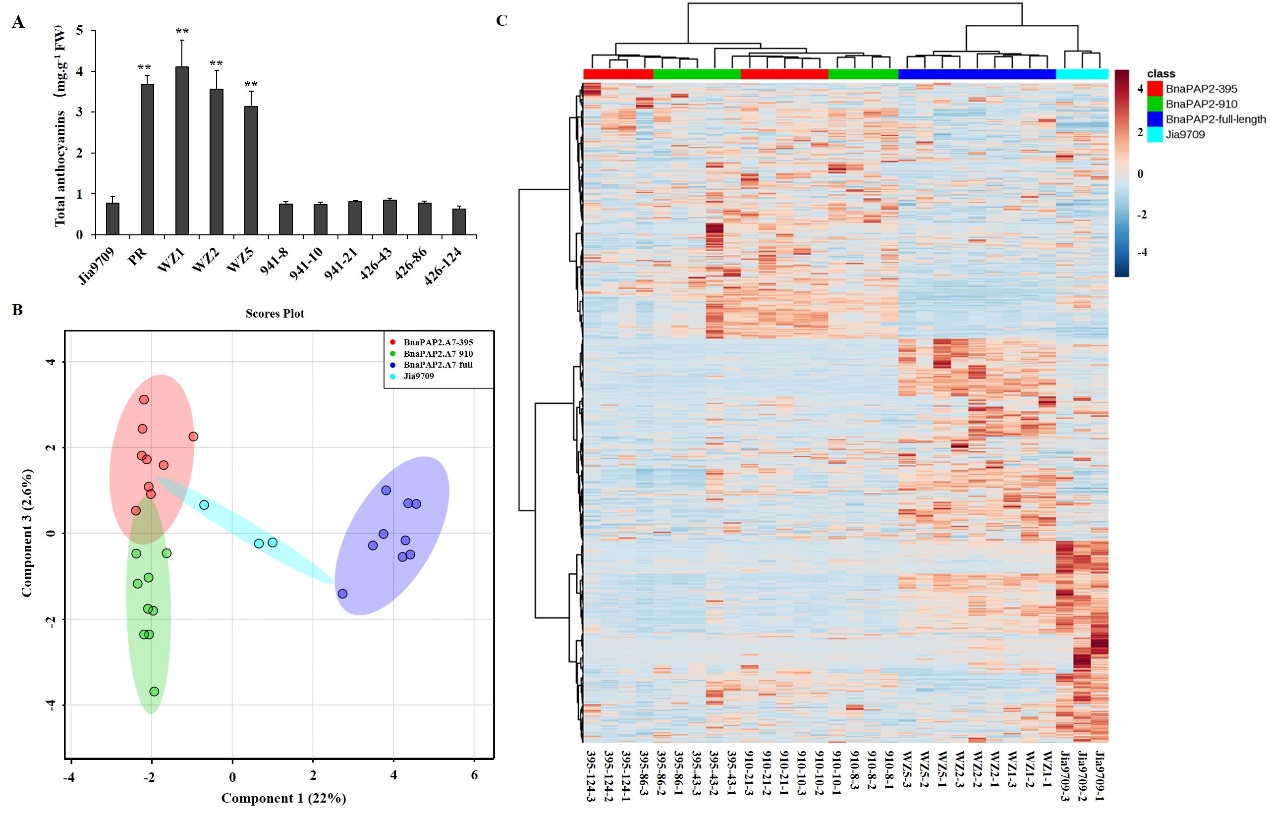


**Figure S1.** Analysis of anthocyanin content and related metabolites in transgenic lines (A) Analysis of anthocyanin content in leaves of transgenic lines containing full length PR *BnaPAP2. A7*, 395 and 910 isoform driven by the same 1.99kb promoter of *BnaPAP2. A7*. (B) Metabolites based principal component analysis (PCA) of controls, full-length BnaPAP2.A7, 395 and 910 transgenic lines.(C) Heat map of different plants based on the relative content of each metabolite.**, P < 0.01, one-way ANOVA test. Error bars represent the SD of three replicates.


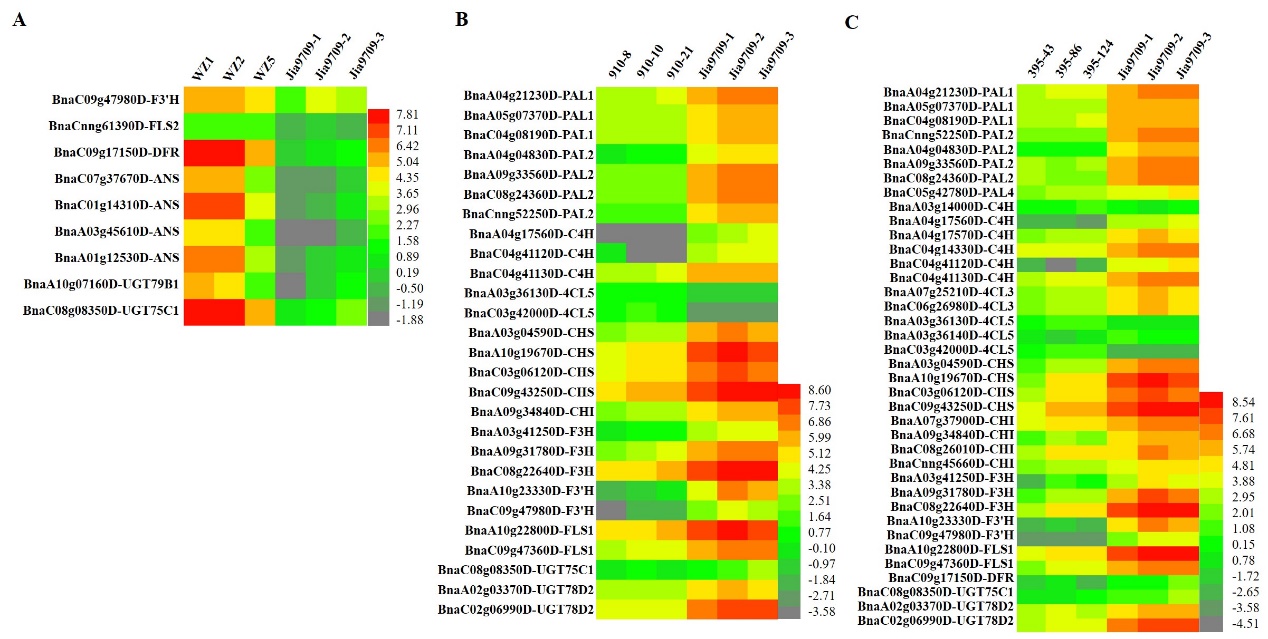


**Figure S2.** Heat map of DEGs involved in anthocyanin biosynthesis pathway

(A-C) DEGs involved in the anthocyanin metabolic pathway in full length *BnaPAP2.A7* (A), 910 (B) and 395 (C) transgenic lines. Note: only structure genes in anthocyanin biosynthesis pathway were presented here.

**Table S1. MBW components identified in *Brassica rapa, B. oleracea and B. napus***

| No. | Gene name | Gene ID | | | | |
| --- | --- | --- | --- | --- | --- | --- |
|  |  | ***Arabidopsis*** | ***B. rapa*** | ***B. oleracea*** | ***B. napus* A subgenome** | ***B. napus* C subgenome** |
| 1 | PAP1(MYB75) | AT1G56650 | BraA02g017040 | Bo00835s060 | BnaA02g35530D | BnaCnng28030D |
| 2 | MYB113 | AT1G66370 | BraA03g040840 | Bo3g081880 | BnaAnng41910D | BnaC03g74080D |
| 3 | MYB114 | AT1G66380 | BraA07g032100 | Bo6g099880 | BnaA07g25800D | BnaCnng69610D |
| 4 | PAP2(MYB90) | AT1G66390 |  | Bo6g100940 |  | BnaCnng17820D |
| 5 |  |  |  |  |  | BnaCnng75540D |
| 6 | EGL3 | AT1G63650 | BraA09g013280 | Bo9g029230 | BnaA09g11090D | BnaC09g11380D |
| 7 |  |  | BraA09g015130 | Bo9g035460 | BnaA09g12420D | BnaC09g12820D |
| 8 | TT8 | AT4G09820 | BraA09g028560 | Bo9g086910 | BnaA09g22810D | BnaC09g24870D |
| 9 | GL3 | AT5G41315 | BraA04g013890 | Bo4g141990 | BnaA04g11060D | BnaCnng33960D |
| 10 | TTG1 | AT5G24520 | BraA02g042110 | Bo7g096780 | BnaA02g32510D | BnaC02g41250D |
| 11 |  |  | BraA06g031870 |  |  | BnaC07g29950D |

**Table S2. Statistics of transformation of *BnaPAP2.A7-744*under different conditions**

| Gene construct/Combinations | Infection time (min） | Infection concentration (OD) | Total explants | Effective callus | Positive transgenic plants containing 744 isoform |
| --- | --- | --- | --- | --- | --- |
| BnaPAP2.A7-744 | 15 | 0.8 | 195 | 0 | 0 |
| BnaPAP2.A7-744 | 30 | 0.8 | 156 | 0 | 0 |
| BnaPAP2.A7-744 | 60 | 0.8 | 29 | 0 | 0 |
| BnaPAP2.A7-744 | 15 | 1.6 | 138 | 0 | 0 |
| BnaPAP2.A7-744 | 15 | 2.4 | 343 | 0 | 0 |
| BnaPAP2.A7-395 | 15 | 0.8 | 726 | 640 | 0 |
| BnaPAP2.A7-744 | 15 | 0.8 | 472 | 0 | 0 |
| BnaPAP2.A7-910 | 15 | 0.8 | 382 | 360 | 0 |
| No Kana-BnaPAP2.A7-744 | 15 | 0.8 | 375 | 366 | 0 |
| BnaPAP2.A7-395 & BnaPAP2.A7-744 | 15 | 0.8 | 519 | 432 | 0 |
| BnaPAP2.A7-744 & BnaPAP2.A7-910 | 15 | 0.8 | 707 | 564 | 0 |
| BnaPAP2.A7-395 & BnaPAP2.A7-744& BnaPAP2.A7-910 | 15 | 0.8 | 885 | 603 | 0 |

**Table S3. Sequences of all primers used in this study**

| Name | Primer sequence (5’―3’) |
| --- | --- |
| Primers for plasmid construction | |
| PAP2.A7-PF | TCATTTTACACATCATCTTCTACTACA |
| PAP2.A7-gR | ACTAATCAAGTTCCAGTTTCTCCA |
| PAP2.A7-CDS-PF | ATGGAGGGTTCGTCCCAAGGG |
| PAP2.A7-CDS-PR | ACTAATCAAGTTCCAGTTTCTCCA |
| PAP2.A7-KpnI-PF | TCGATCTGGTACCTCATTTTACACATCATCTTCTACTACA |
| PAP2.A7-BamHI-gR | AGTACAGGATCCACTAATCAAGTTCCAGTTTCTCCA |
| PAP2.A7-BamHI-PR | AGTACAGGATCC GGACCAGCTATAATTTTAGAAGTA |
| PAP2.A7-BamHI-PF | TAAATTGGTACCATGGAGGGTTCGTCCCAAGGG |
| PAP2.A7-BstEII-PR | CAGTCAGGTCACCACTAATCAAGTTCCAGTTTCTCCA |
| Primers for yeast two hybrid | |
| AtPAP2-F | ATGGAGGGTTCGTCCAAAGGG |
| AtPAP2-R | CTAATCAAGTTCAACAGTCTCTC |
| AtPAP2-EcoRI-F | AGTGCAGAATTCATGGAGGGTTCGTCCAAAGGG |
| AtPAP2-BamHI-R | AGTACAGGATCCCTAATCAAGTTCAACAGTCTCTC |
| SG -EcoRI-F | AGTGCAGAATTCATGGAGGGTTCGTCCCAAGGG |
| SG-BamHI-R | AGTACAGGATCCTCAAGTTCCAGTTTCTCCATCCAA |
| At-TT8-F2 | GAGAGAGAGCTACCACGTTTT |
| At-TT8-R2 | TGGCATCAATAAAGTTAGGGT |
| AtTT8-EcoRI-F | AGTGCAGAATTCATGGATGAATCAAGTATTATT |
| AtTT8-BamHI-R | AGTACAGGATCCCTATAGATTAGTATCATGTAT |
| Primers for subcellular localization | |
| A7-Eco105I-PF | TAAATTTACGTACCATGGAGGGTTCGTCCCAAG |
| A7-SmiI-PR | CGCCGGTAAATTTAACTAATCAAGTTCCAGTTTCTCCA |
| AT-BamHI-PF | AGTACAGGATCCCTTTTATATTAATTTTTCTTTGGTCC |
| AT-SmaI-PR | AGTACACCCGGGCTTAAACAAACGGTGAGAAAC |
| Primers for qRT-PCR | |
| Bnaactin3-eF | TCCATCCATCGTCCACAG |
| Bnaactin3-eR | GCATCATCACAAGCATCCTT |
| RTPAP2A7-3-F | GCATTGATAAGTATGGAGAAGG |
| RTPAP2A7-426-R5 | CCTATTCTGTTTCCTAAAAGC |
| RTPAP2A7-775-R3 | GACCACCTGTTTCCTAAAAGC |
| RTPAP2A7-941-R | GGGATATATTTGACTTAGTAATGG |
| PAL-A04-RT1F | GTCTTCCTTCGAACCTAACC |
| PAL-A04-RT1R | TGAGTATGTCTACAGCTTCTGAT |
| C4H-C04-RT1F | CTATTGGTAGGTTGGTTCAAAACTTT |
| C4H-C04-RT1R | TGCAGAAATCATATTTTCTTGTCTATCTT |
| CHS-A10-RT1F | TCCTTGACGAGGTTGAGAAG |
| CHS-A10-RT1R | CAAGACCACTGTCTCTACG |
| F3'H-A10-RT1F | TGCACGGATTTGAATGGGAACT |
| F3'H-A10-RT1R | GCTTGTCATGCAAGCTAAGTTAG |
| DFR-C09-RT1F | TGTTCGCGATCCTGGAAATTTGA |
| DFR-C09-RT1R | CTTTGATTCAAAATCCATGGGAGTA |
| ANS-A01-RT1F | TGTTTTGTGAACCACCAAAGGAT |
| ANS-A01-RT1R | CTCAAACATAGAGTTTCAGACTCA |
| MYB4-C03-RT1F | ACAACACCATTAACATATCTTTCACTAA |
| MYB4-C03-RT1R | CTAAACTGCACTTGAAACAACGCT |
| MYBL2-A02-RT1F | AGTTTGTGGCCTTCAAGAATCTAA |
| MYBL2-A02-RT1R | TACAGATAAATACACCATTTTTCA |
